# Inhibition of yes‐associated protein suppresses brain metastasis of human lung adenocarcinoma in a murine model

**DOI:** 10.1111/jcmm.13582

**Published:** 2018-03-24

**Authors:** Ping‐Chih Hsu, Jinbai Miao, Zhen Huang, Yi‐Lin Yang, Zhidong Xu, Joanna You, Yuyuan Dai, Che‐Chung Yeh, Geraldine Chan, Shu Liu, Anatoly Urisman, Cheng‐Ta Yang, David M. Jablons, Liang You

**Affiliations:** ^1^ Department of Surgery Helen Diller Family Comprehensive Cancer Center University of California San Francisco CA USA; ^2^ Department of Thoracic Medicine Chang Gung Memorial Hospital Linkou Taoyuan Taiwan; ^3^ Department of Thoracic Surgery Beijing Chao‐Yang Hospital Affiliated with Capital Medical University Beijing China; ^4^ Department of Hepatobiliary Surgery National Cancer Center/Cancer Hospital Chinese Academy of Medical Sciences and Peking Union Medical College Beijing China; ^5^ Department of Pathology University of California San Francisco CA USA

**Keywords:** Hippo signalling, K‐ras mutation, lung adenocarcinoma, metastasis, yes‐associated protein

## Abstract

Yes‐associated protein (YAP) is a main mediator of the Hippo pathway and promotes cancer development and progression in human lung cancer. We sought to determine whether inhibition of YAP suppresses metastasis of human lung adenocarcinoma in a murine model. We found that metastatic NSCLC cell lines H2030‐BrM3(K‐ras^G12C^ mutation) and PC9‐BrM3 (EGFR ^Δexon19^ mutation) had a significantly decreased p‐YAP(S127)/YAP ratio compared to parental H2030 (K‐ras^G12C^ mutation) and PC9 (EGFR ^Δexon19^ mutation) cells (*P* < .05). H2030‐BrM3 cells had significantly increased YAP mRNA and expression of Hippo downstream genes CTGF and CYR61 compared to parental H2030 cells (*P* < .05). Inhibition of YAP by short hairpin RNA (shRNA) and small interfering RNA (siRNA) significantly decreased mRNA expression in downstream genes CTGF and CYR61 in H2030‐BrM3 cells (*P* < .05). In addition, inhibiting YAP by YAP shRNA significantly decreased migration and invasion abilities of H2030‐BrM3 cells (*P* < .05). We are first to show that mice inoculated with YAP shRNA‐transfected H2030‐BrM3 cells had significantly decreased metastatic tumour burden and survived longer than control mice (*P* < .05). Collectively, our results suggest that YAP plays an important role in promoting lung adenocarcinoma brain metastasis and that direct inhibition of YAP by shRNA suppresses H2030‐BrM3 cell brain metastasis in a murine model.

## 1 INTRODUCTION

Lung adenocarcinoma accounts for approximately 40% of primary lung cancer worldwide, and in most patients, the disease is already metastatic at the time of their initial diagnosis.^1^, ^2^ Metastasis in lung adenocarcinoma is a major morbidity that is associated with poor prognosis and an overall 5‐year survival rate less than 7%.^3^, ^4^, ^5^, ^6^ Despite the developments of drugs targeting specific cancer‐driven mutations, treatment options for metastatic lung adenocarcinoma without targetable mutations remain quite limited. For example, the epidermal growth factor receptor (EGFR)‐tyrosine kinase inhibitor (TKI) erlotinib is an effective target therapy for metastatic lung adenocarcinoma harbouring EGFR mutations such as exon 19 deletion or exon 21 L858R mutations. Erlotinib had a better response rate and fewer side effects than conventional chemotherapy in treating patients with EGFR mutant metastatic lung adenocarcinoma.^7^, ^8^, ^9^, ^10^ K‐ras mutation is frequent in patients with lung adenocarcinoma (15%‐30%), but there is still no approved effective target therapy for K‐ras mutant metastatic lung adenocarcinoma.^10^, ^11^, ^12^

Yes‐associated protein (YAP), a key mediator protein in the Hippo (also known as the Salvador‐Warts‐Hippo) signalling pathway, has been reported to promote development of various cancers.^13^, ^14^, ^15^ YAP has been suggested as a potential therapeutic target for melanoma, mesothelioma and hepatocellular carcinoma.^16^, ^17^, ^18^ YAP was also identified in human non‐small‐cell lung cancer (NSCLC) and is correlated with drug resistance and tumorigenesis.^14^, ^19^, ^20^, ^21^ A previous study reported that because some NSCLC cell lines harbouring K‐ras mutations were not K‐ras‐dependent cells, K‐ras may be not an appropriate therapeutic target.^22^ Furthermore, YAP appears to take over K‐ras as a cancer driver in NSCLC and pancreatic ductal adenocarcinoma, and YAP was identified as a central driver of compensation for K‐ras‐dependent NSCLC when there is loss of K‐ras signalling.^23^, ^24^, ^25^ Metastatic lung adenocarcinoma cell line H2030‐BrM3 (K‐ras^G12C^ mutation) was derived from lung adenocarcinoma cell line H2030 (K‐ras^G12C^ mutation), which has a known cancer‐driven K‐ras mutation and a high potential of metastasis in murine models.^26^, ^27^ Although an important regulator, serpin I1 (neuroserpin), was identified in H2030‐BrM3 cells to promote brain metastasis, rare study found effective treatments for metastatic K‐ras mutant lung adenocarcinoma in murine models.^27^

We sought to determine whether there is increased Hippo/YAP signalling pathway reporter activity and YAP expression in metastatic lung adenocarcinoma cells. In addition, we investigated whether direct inhibition of YAP suppresses human lung adenocarcinoma H2030‐BrM3 cell metastasis in vivo.

## 2 MATERIALS AND METHODS

### 2.1 Cell culture

Human metastatic NSCLC cell lines H2030‐BrM3 (K‐ras^G12C^ mutation) (P9) and PC9‐BrM3 (EGFR^Δexon19^ mutation) (P40) were kindly provided by Professor Joan Massagué (Metastasis Research Center, Memorial Sloan Kettering Cancer Center, New York, NY) and authenticated by left ventricle inoculation as previously described.^26^, ^27^ Parental human NSCLC cell line PC9 (P10) was a gift from Dr. Jasmine G. Lee (Department of Internal Medicine, Division of Respiratory Medicine, University of California Davis, Davis) and authenticated by erlotinib treatment in a viability assay.^28^ Human NSCLC cell line H2030 was obtained from American Type Culture Collections (ATCC) (Manassas, VA) and passaged for fewer than 6 months after receipt from ATCC. Cell lines were maintained in RPMI‐1640 supplemented with 10% heat‐inactivated foetal bovine serum and streptomycin (100 mg/mL), and were cultured at 37°C in a humid incubator with 5% CO_2_.

### 2.2 Animal studies

All in vivo experiments strictly followed the UCSF institutional guidelines (Institutional Animal Care and Use Committee approval number: AN103205‐03). Athymic nude (CrTac:NCr‐Foxn1nu) female mice, 6‐8 weeks of age, were purchased from Taconic Farms, Inc (Hudson, NY 12534). A metastatic murine model was created by injecting 500 000 H2030‐BrM3, YAP shRNA‐transfected H2030‐BrM3 or control shRNA‐transfected H2030‐BrM3 cells suspended with 100 μL of PBS into the left ventricle via a percutaneous approach as previously described.^26^, ^27^ The mice were killed when they showed symptoms of paralysis or appeared extremely sick, according to the guidelines established by the UCSF Laboratory Animal Resource Center, and tumour tissues were collected.

Bioluminescent imaging (Caliper Life Sciences, Waltham, MA) was used to monitor in vivo metastasis. D‐luciferin potassium salt (Syd Labs, Natick, MA) was injected intraperitoneally into the mice at a dose of 150 mg/kg, and mice were anaesthetized by inhalant isoflurane 1%‐5% in oxygen. After 10 minutes, the mice were placed into the Xenogen IVIS spectrum imaging system. Images were recorded with an exposure time of 1 minute. Survival was measured from the day of H2030‐BrM3 cell injection to the day mice were killed.

### 2.3 Cell viability assay

Parental PC9 and PC9‐BrM3 cells were cultured in a 96‐well plate and treated with different doses of erlotinib (Selleckchem; Houston, TX) (0, 0.01, 0.03, 0.1, 0.3, 1, 3, 10, 30, 100 μmol/L). H2030‐BrM3 cells transfected with control, YAP short hairpin RNA (shRNA) and YAP shRNA‐2 were cultured in a 96‐well plate. After 72 hours of incubation, cells were lysed and CellTiter‐Glo Luminescent Cell Viability Assay reagent (Promega) was added to generate luminescent signalling. Luminescent signalling was detected using the GloMax‐96 Microplate Luminometer. GraphPad Prism 5.0 software (GraphPad Software, Inc., La Jolla, CA) was used to analyse proportional cell viability and calculate dose‐response curves and IC50.

### 2.4 Short hairpin RNA transfection and cell selection

Yes‐associated protein shRNA#1 and control shRNA lentiviral particles were purchased from Santa Cruz Biotechnology, Inc (Dallas, TX). YAP shRNA#2 lentiviral particles were purchased from Sigma‐Aldrich (St. Louis, MO) and had a different targeting sequence from YAP shRNA#1. H2030‐BrM3 cells were placed into 12‐well plates and grown to 70% confluence. The particles were mixed with transfection medium and reagents according to the manufacturer's protocol and then added to the cells. Bulk selection by adding puromycin or geneticin (Life Technologies Corporation) in culture medium was started at day 3 after transfection. Puromycin at a concentration of 2.5 μg/mL and mixed with culture medium was used for YAP shRNA#1‐ and control shRNA‐transfected cell selection. Geneticin at a concentration of 500 μg/mL mixed with culture medium was used for YAP shRNA#2‐transfected cell selection. The duration from the beginning of puromycin or geneticin selection to shRNA‐transfected cell ready for intracardiac injection was at least 2 months. The efficacy of transfection was confirmed by Western blotting.

### 2.5 Luciferase gene, small interfering RNA and cDNA plasmid transfection

For luciferase gene transfection, the pGreenFire1‐CMV Virus (pTRH1 CMV dscGFP T2A Fluc) Positive Control was purchased from System Bioscience LLC (Palo Alto, CA). Virus particles were mixed with transfection reagents and medium according to the manufacturer's protocol and added to the H2030‐BrM3, YAP shRNA‐transfected and control shRNA‐transfected H2030‐BrM3 cells cultured in 12‐well plates. The efficacy of transfection was determined by fluorescence microscopy.

EGFR small interfering RNA (siRNA) and K‐ras siRNA were purchased from Santa Cruz Biotechnology, Inc (Dallas, TX). The SMARTPool siRNA that targets YAP was purchased from Dharmacon (Pittsburgh, PA), and the targeting sequence was different from YAP shRNA#1 and YAP shRNA#2. Cells were transfected with 100 nmol/L of siRNA using Lipofectamine RNAiMAX (Invitrogen, Carlsbad, CA).

The YAP plasmid DNA used to overexpress the YAP gene in the cells was purchased from Addgene (Cambridge, MA). Cells were placed in 6‐well plates and transfected with 4 μg of YAP plasmid DNA using Lipofectamine 2000 transfection reagent.

### 2.6 Western blot analysis

The total amount of protein for each sample was 20 μg, and the samples were run on 4%‐20% gradient SDS‐polyacrylamide gels (Bio‐Rad Laboratories, Inc., Hercules, CA) and then were transferred to immobilon‐P nitrocellulose membranes (Millipore, Bellerica, MA). The membranes were probed with rabbit anti‐YAP (Cell Signaling, 4912; 1:1000), rabbit anti‐phospho‐Yap Ser127 (Cell Signalling, 9411; 1:1000), mouse antineuroserpin(serpin I1) (Abcam, ab125097; 1:500) and mouse anti‐GAPDH (Sigma‐Aldrich, 100242‐MM05; 1: 10 000) in 4°C overnight after being blocked with 5% non‐fat milk. The membranes were then incubated with species‐specific‐conjugated secondary antibodies (GE Dharmacon) at room temperature for 1 hour. An ECL blotting analysis system (Amersham Pharmacia Biotech, Piscataway, NJ) was used for detecting protein expression.

### 2.7 Luciferase reporter assay

The control shRNA‐transfected H2030‐BrM3 and YAP shRNA‐transfected H2030‐BrM3 cells were placed in 24‐well plates and transfected with 8× GTIIC‐luciferase plasmid (Addgene, Cambridge, MA) and Renilla luciferase pRL‐TK plasmid (Promega, Madison, WI) using transfection reagent Lipofectamine 2000 (Invitrogen, Carlsbad, CA). After 48‐hour transfection, cells were lysed and luciferase activity was assayed using Dual‐Luciferase Reporter Assay system (Promega, Madison, WI). All luciferase activities were normalized to Renilla activity.

### 2.8 DNA, RNA isolation, cDNA synthesis and quantitative real‐time RT‐PCR

The High Pure RNA Isolation Kit (Roche, Indianapolis, IN) was used for total RNA extraction from cells, and the QIAamp DNA Mini Kit (Qiagen, Valencia, CA) was used for DNA extraction from cells. The iScript cDNA Synthesis Kit (Bio‐Rad, Hercules, CA) was used to transcribe RNA to the cDNA, and the cDNA was used as the template for real‐time PCR. Real‐time PCR detection was using TaqMan Technology on an Applied Biosystems 7000 Sequence Detection System (Applied Biosystems). Primers and probe sequences commercially available (Applied Biosystems, Foster City, CA) were used to detect YAP, CYR61, CTGF, serpin I1 and endogenous control gene b‐glucuronidase (GUSB) gene expression. Relative Quantification Software (Applied Biosystems) was used for mRNA expression analysis. Quantitative PCR for YAP copy number was assayed by TaqMan^®^ Copy Number Assays, and the result was analysed using CopyCaller^®^ Software v2.0 (Thermo Fisher Scientific, Waltham, MA).

### 2.9 Wound‐healing and transwell invasion assays

Parental H2030‐BrM3, YAP shRNA#1‐transfected, control shRNA‐transfected H2030‐BrM3 cells were sub‐cultured in 6‐well plates to the condition of confluence. The plates were scratched by a 200‐μL pipette tip, and the cells were grown continuously. Phase contrast images were taken at the time of the scratch (0 hour) and after 18 hours. The distance of cell movement was measured by ImageJ software.

The 6‐well plate transwell system (Corning Incorporated, USA) was used for the transwell invasion assay, and the transwell inserts were coated with 300 μL Matrigel. YAP shRNA‐transfected and control shRNA‐transfected H2030‐BrM3 cells were trypsinized and resuspended in serum‐free medium, and the cells were seeded on the upper chamber of the transwell. The lower chamber was infused with 2 mL complete growth medium (10% FBS). The gel and cells in the upper chamber of the transwell were wiped after incubation at 37°C for 48 hours. The membrane was stained by crystal violet for 10 minutes after methanol fixation. Phase contrast images were taken with a Primo Vert microscope (Zeiss, Gottingen, Germany), and the cells on the lower side of the membrane were counted.

### 2.10 YAP immunofluorescence stain

H2030, H2030‐BrM3 and YAP shRNA#1‐transfected H2030‐BrM3 cells were sub‐cultured in an 8‐well chamber slide (Thermo Fisher Scientific, Waltham, MA). The cells were fixed with 100% methanol the next day. Cells were blocked in 5% goat serum for 1 hour, rabbit anti‐YAP primary antibody (Cell Signaling, 14074; 1:100) was added, and cells were incubated in 4°C overnight. Goat anti‐rabbit IgG (H+L) for secondary antibody with Alexa Fluor^®^ 488 (Thermo Fisher Scientific, A‐11008; 1:800) for primary antibody detection were added, and cells were incubated at room temperature for 1 hour. The cells were then washed with PBS 3 times, and nuclei were stained by 2 μg/mL DAPI in PBS. A fluorescence microscope (Zeiss Axioskop 2 microscope; Carl Zeiss, Inc., Germany) was used to view stained cells, and images were taken under a 20× objective lens.

### 2.11 Tissue samples, histologic analysis and immunohistochemistry

After mice were killed, the tumour tissues from metastatic sites including lung, bone and brain were harvested and immediately fixed in 10% paraformaldehyde for 24 hours. The tissues were then transferred into 70% ethanol for storage and embedded in paraffin. Histological sections of the tissues were stained with haematoxylin and eosin (H&E stain) for general morphology analysis. An experienced pathologist did the morphological analysis in the stained tissues to verify tumour metastasis. Rabbit anti‐YAP antibody (Cell Signaling, 4912; 1:500) used for immunohistochemistry (IHC) staining was from Cell Signaling Technology. The brain tissues sections of control‐ and YAP shRNA‐transfected H2030‐BrM3 intracardiac‐injected mice were immunostained. The brain tissue slides underwent routine deparaffinization and rehydration. Slides were then immersed in boiled 10 mmol/L sodium citrate buffer (pH 6.0), for 10 minutes. After cooling down, sections were incubated in 3% hydrogen peroxide for 10 minutes and then washed in PBS three times for 5 minutes. Sections were incubated with 10% normal horse serum in PBS for 30 minutes. After being washed in PBS three times, the sections were incubated overnight at 4°C with rabbit anti‐YAP antibody (Cell Signaling, 4912; 1:500). The next day, the sections were incubated with biotin‐labelled secondary antibodies and streptavidin‐peroxidase (1: 30) for 20 minutes each. Slides were stained for 5 minutes with 0.05% 3,3′‐diaminobenzidine tetrahydrochloride freshly prepared in 0.05 mol/L Tris‐HCl buffer (pH 7.6) containing 0.024% hydrogen peroxidase. The slides then were counterstained with haematoxylin, dehydrated and mounted in Diatex. All images were taken by a Zeiss Axioskop 2 microscope (Carl Zeiss Inc., Germany).

### 2.12 ChIP assay

Chromatin ImmunoPrecipitation (ChIP) Assay Kit (Millipore Corporation) was used for the ChIP assay. Polyclonal antibodies for YAP and control rabbit antibody for IgG used for ChIP were purchased from Cell Signaling Technology.

Primers used for RT‐PCR to amplify the neuroserpin(serpin I1) gene were 5′‐ATAGTGCCTCTACCTCGAAG‐3′ and 5′‐TACTTAGGCTGGTCCCTTGG‐3′, resulting in a product size of 250 bp.

### 2.13 Statistical analysis

Data are expressed as mean ± standard deviation (SD) from three independent experiments. All statistical analyses were performed with GraphPad Prism (version 5.0; GraphPad Software, San Diego, CA, USA). *t* Tests were used to compare the differences between two groups, and one‐way ANOVA followed by Tukey's multiple comparisons was used to compare differences among >2 groups. A Kaplan‐Meier survival curve was calculated to determine survival in the animal experiments and patients in tissue microarray. All *P* values were 2‐sided and considered to be statistically significant if *P* was less than .05 (*P < .05, **P < .01, ***P < .001).

## 3 RESULTS

### 3.1 P‐YAP(S127)/YAP ratio is decreased in metastatic NSCLC cell lines

**Figure 1:**
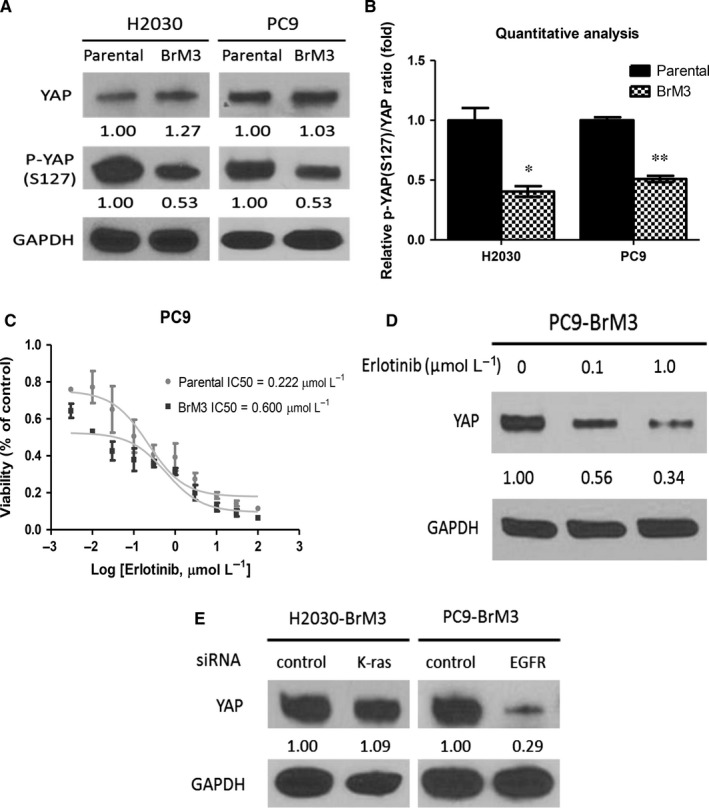
Metastatic non‐small‐cell lung cancer cell lines have decreased p‐YAP(S127)/YAP ratio compared to their parental cell lines. A, Western blot analysis of YAP and p‐YAP(S127) protein expression in H2030‐BrM3, parental H2030, PC9‐BrM3 and parental PC9 cells. B, Quantitative analysis of p‐YAP (S127)/YAP protein expression ratio in H2030‐BrM3, parental H2030, PC9‐BrM3 and parental PC9‐BrM3 cells. C, Cell viability analysis in PC9 and PC9‐BrM3 cells after erlotinib treatment. D, YAP protein decreased after 0.1 and 1.0 μmol/L erlotinib treatments in PC9‐BrM3 cells. E, YAP protein expression in H2030‐BrM3 cells after K‐ras knockdown and in PC9‐BrM3 cells after EGFR knockdown (error bars indicate standard deviations; *P < .05 and **P ≤ .01)

To investigate whether metastatic NSCLC cell lines have increased YAP stability, Western blotting of YAP and p‐YAP(S127) was assayed. Western blotting showed that p‐YAP(S127)/YAP ratio was decreased in the metastatic NSCLC cell lines H2030‐BrM3 (K‐ras^G12C^ mutation) and PC9‐BrM3 (EGFR^Δexon19^ mutation) compared to parental H2030 and PC9 cell lines (*P* < .05) (Figure 1A,B).

When the cell viability of PC9‐BrM3 and parental PC9 cells treated by erlotinib was assayed, we found that the IC50 of erlotinib was 0.600 μmol/L for PC9‐BrM3 cells and 0.222 μmol/L for parental PC9 cells (Figure 1C). In PC9‐BrM3 cells, YAP protein expression decreased after dose‐dependent erlotinib treatment (Figure 1D). Western blotting showed that YAP protein expression did not change in K‐ras siRNA‐transfected H2030‐BrM3 cells and that YAP protein expression was decreased in EGFR siRNA‐transfected PC9‐BrM3 cells (Figure 1E).

The finding that p‐YAP(S127)/YAP ratio decreased in metastatic NSCLC cell lines indicates that YAP stability increased. In the EGFR mutant cell line PC9‐BrM3 (EGFR^Δexon19^ mutation), erlotinib treatment decreased YAP protein expression. In K‐ras mutant H2030‐BrM3 cells (K‐ras^G12C^ mutation), K‐ras knockdown by K‐ras siRNA did not decrease YAP protein expression.

### 3.2 YAP activation originates at the transcription level in the metastatic NSCLC cell line H2030‐BrM3

**Figure 2:**
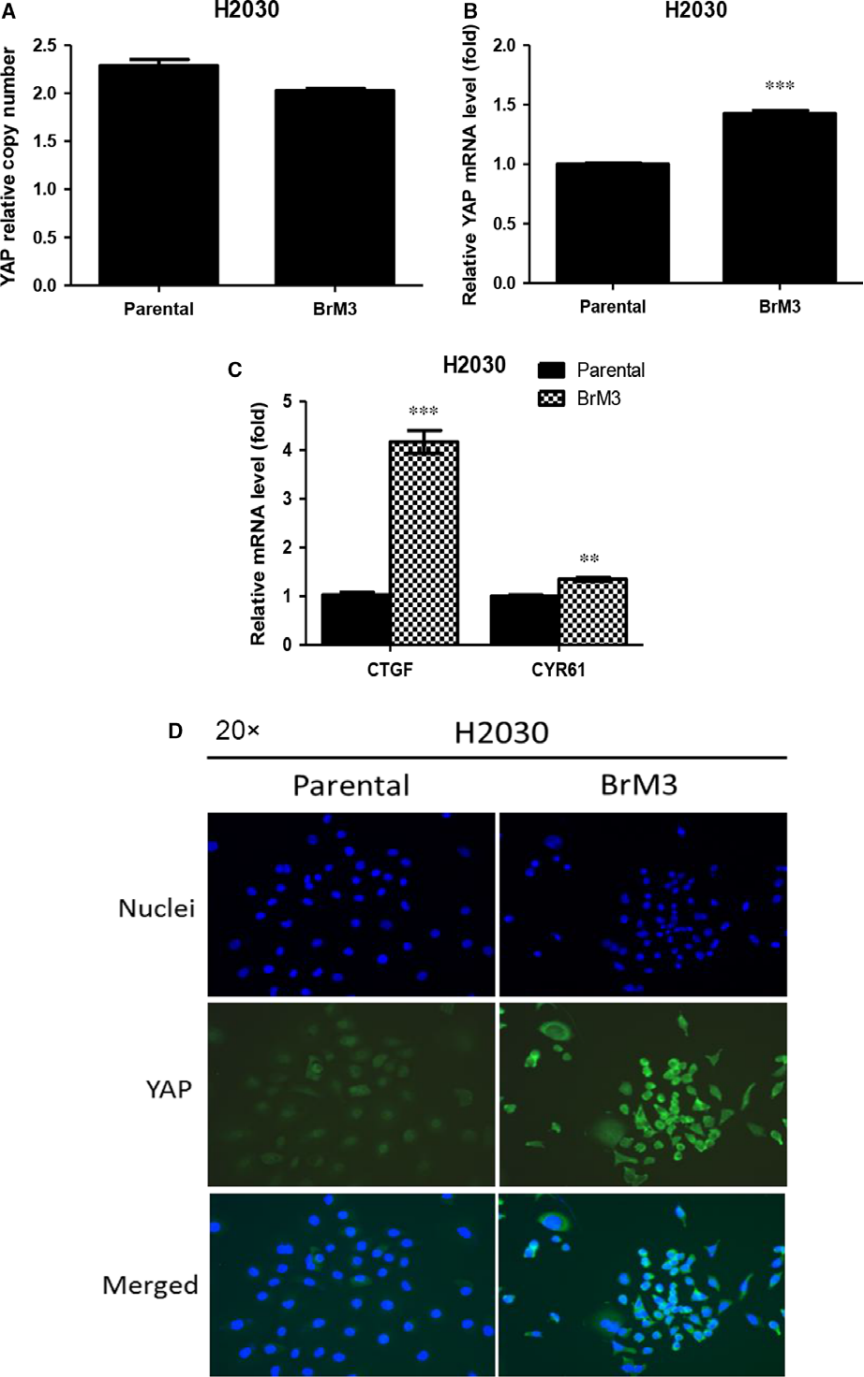
H2030‐BrM3 cells have increased YAP mRNA, expression of downstream genes CTGF and CYR61, and YAP immunofluorescence staining compared to parental H2030 cells. A, QPCR for YAP DNA copy number analysis showed that parental H2030 and H2030‐BrM3 cells have two copies of YAP. B, YAP mRNA expression level significantly increased in H2030‐BrM3 cells. C, mRNA expression of the Hippo downstream genes CTGF and CYR61 mRNA expressions significantly increased in H2030‐BrM3 cells. D, Immunofluorescence stain assay showed that H2030‐BrM3 cells had increased YAP staining compared to parental H2030 cells (error bars indicate standard deviations; **P ≤ .01; and ***P ≤ .001)

Quantitative PCR analysis of DNA copy number showed that parental H2030 and H2030‐BrM3 cells had two copies of YAP (Figure 2A). YAP mRNA expression and that of the downstream genes CTGF and CYR61 significantly increased in H2030‐BrM3 compared to parental H2030 cells (*P* < .01) (Figure 2B,C). Finally, immunofluorescence staining showed that H2030‐BrM3 cells had increased YAP staining compared to parental H2030 cells (Figure 2D). Collectively, these findings indicate that YAP activation originates at the transcription level in the H2030‐BrM3 cell line.

### 3.3 Inhibition of YAP decreased expression of downstream genes CTGF and CYR61 in H2030‐BrM3 cells

**Figure 3:**
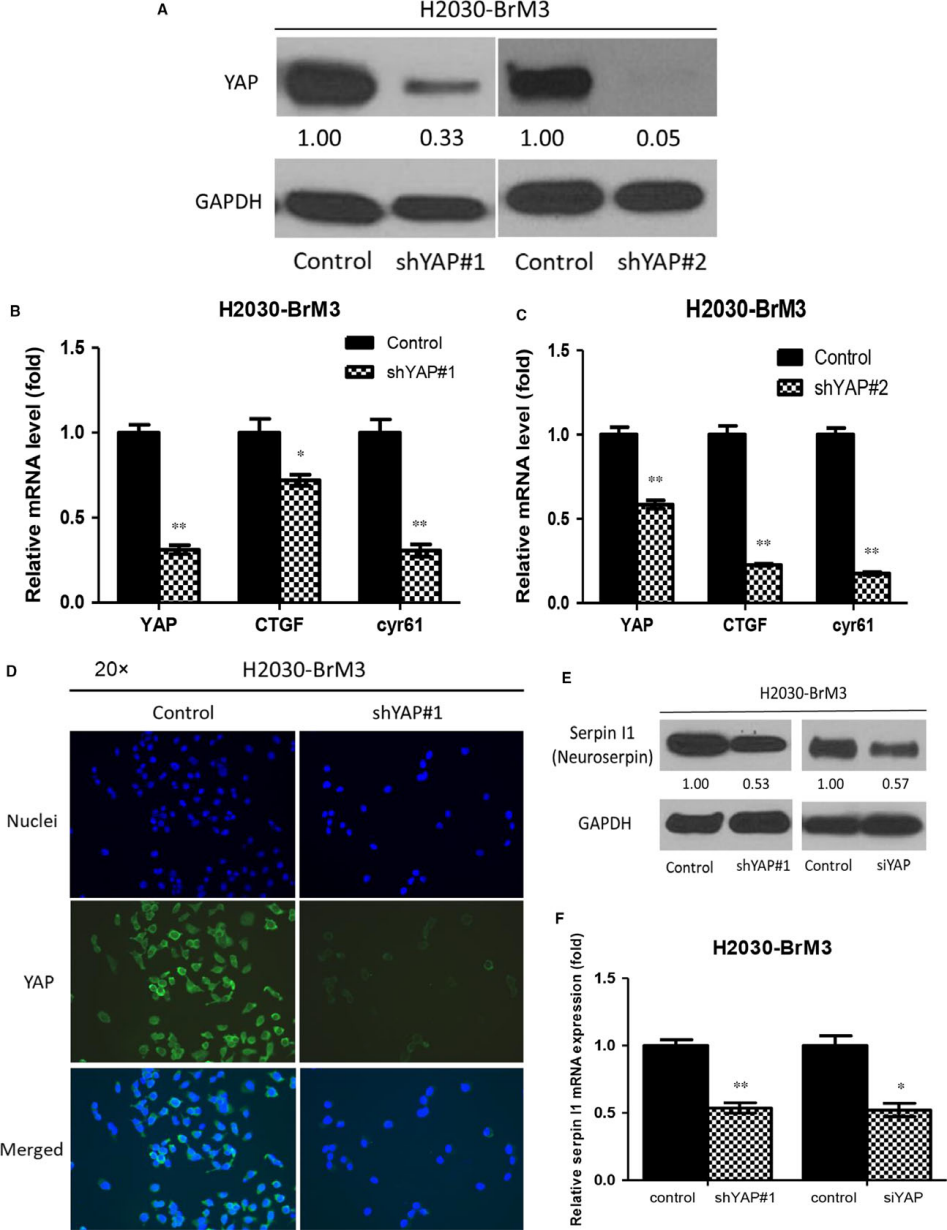
Downstream gene and metastatic regulator expression changes in H2030‐BrM3 cells after YAP knockdown. A, YAP knockdown by siRNA and shRNA decreased YAP protein expression in H2030‐BrM3 cells. B, C, YAP mRNA and mRNA expression of Hippo downstream genes CTGF and CYR61 significantly decreased in YAP shRNA‐transfected H2030‐BrM3 cells. D, Immunofluorescence stain assay showed that YAP staining decreased in YAP shRNA#1‐transfected H2030‐BrM3 cells. E, YAP knockdown by siRNA and shRNA decreased serpin I1 protein expression in H2030‐BrM3 cells. F, YAP knockdown by shRNA and siRNA significantly decreased serpin I1 mRNA expression in H2030‐BrM3 cells (error bars indicate standard deviations; *P < .05 and ***P ≤ .001)

After YAP knockdown by siRNA and shRNA in H2030‐BrM3 cells, we found decreases in YAP protein expression (Figures 3A and S3A), GTIIC reporter activity (Figure S3C), YAP mRNA expression and the transcription of Hippo pathway downstream genes CTGF and CYR61 (*P* < .05) (Figures 3B,C and S3B), and immunofluorescence staining of YAP staining (Figure 3D).

Real‐time PCR showed a significant threefold increase in mRNA expression of the metastatic regulator serpin I1 (neuroserpin) in H2030‐BrM3 cells compared to parental H2030 cells (Figure S1A). Sequence analysis of serpin I1 revealed four putative YAP binding sites between −7372 and −6009 nucleotides upstream of the transcription start site (Figure S1B). ChIP assays performed in H2030‐BrM3 cells demonstrated that YAP binds to the serpin l1 (neuroserpin) enhancer (Figure S1C). To investigate whether inhibition of YAP decreased the expression of serpin I1, protein expression was assayed by Western blotting. Real‐time PCR showed that in H2030‐BrM3 cells, inhibiting YAP by siRNA and shRNA decreased serpin I1 protein expression and decreased serpin I1 mRNA gene expression (*P* < .05) (Figure 3E,F).

Collectively, these results show that inhibition of YAP decreased expression of Hippo pathway downstream genes. We further found that YAP binds to the metastatic regulator serpin l1 promoter, and inhibition of YAP decreased serpin l1 expression at the protein and mRNA level.

### 3.4 Inhibition of YAP by shRNA restrains migration and invasion abilities in H2030‐BrM3 cells

**Figure 4:**
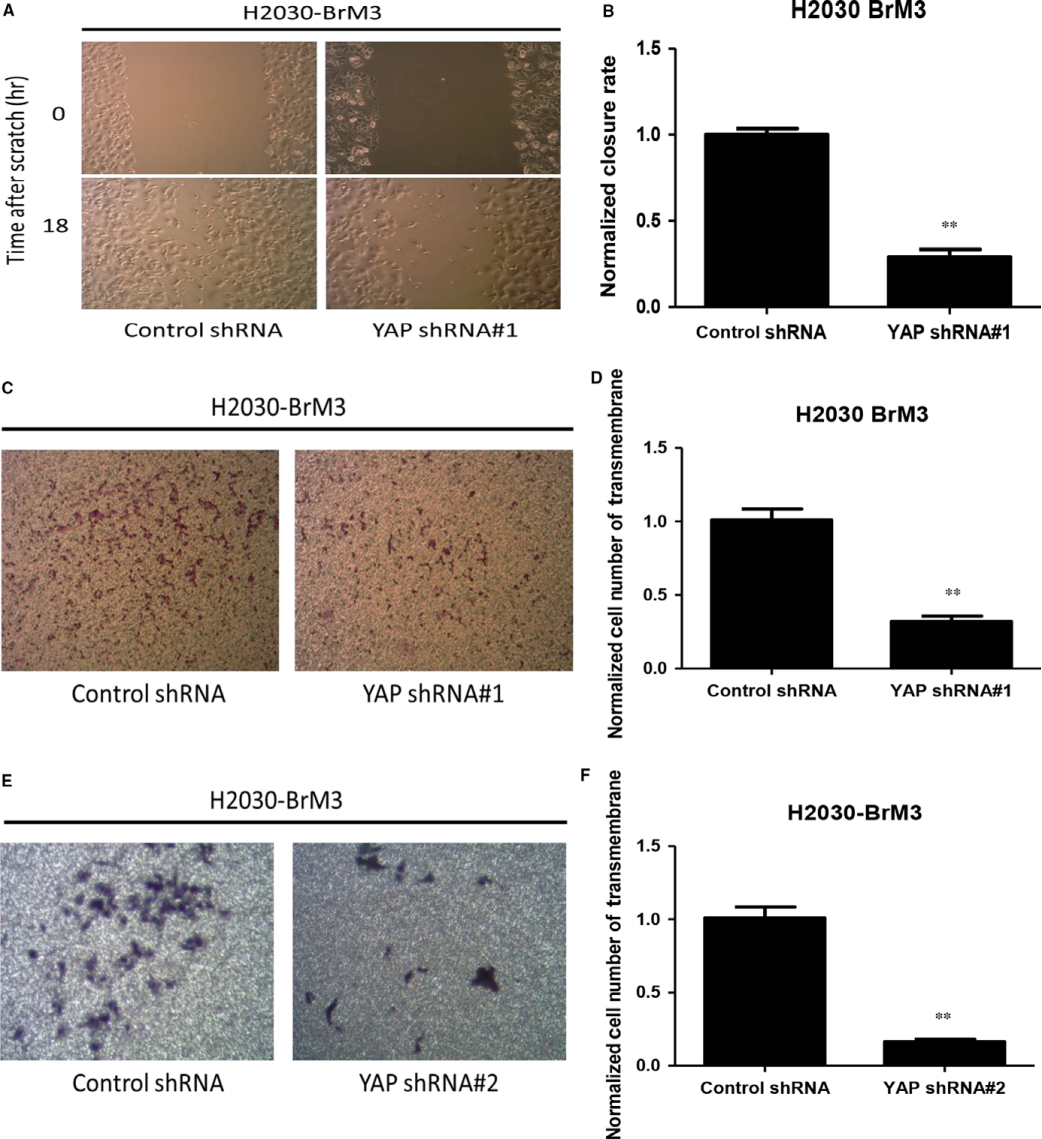
Analysis of cell migration and invasion abilities of H2030‐BrM3 cells after inhibiting YAP by YAP shRNA. A, Decrease in cell migration ability in H2030‐BrM3 cells after YAP knockdown by YAP shRNA#1. B, Quantitative analysis of migration assay result, indicating YAP knockdown by YAP shRNA#1 decreased cell migration ability in H2030‐BrM3 cells. C, Decrease in cell invasion ability in H2030‐BrM3 cells after YAP knockdown by YAP shRNA#1. D, Quantitative analysis of transwell invasion assay result, indicating YAP knockdown by YAP shRNA#1 decreased cell invasion ability in H2030‐BrM3 cells. E, Decrease in cell invasion ability in H2030‐BrM3 cells after YAP knockdown by YAP shRNA#2. F, Quantitative analysis of transwell invasion assay result, indicating YAP knockdown by YAP shRNA#2 decreased cell invasion ability in H2030‐BrM3 cells (error bars indicate standard deviations; **P ≤ .01)

To investigate whether inhibition of YAP restrains migration and invasion abilities in H2030‐BrM3 cells, control shRNA‐transfected and YAP shRNA#1‐transfected H2030‐BrM3 cells were cultured to nearly confluence, scratched with a 200‐μL pipette tip, and after 18 hours, wound closure was measured. The wound closure rate decreased significantly in the transfected cells (Figure 4A,B). The transwell assay showed that the number of the cells that invaded the lower side of the membrane decreased significantly in the YAP shRNA#1‐transfected cells (*P* < .01) (Figure 4C,D). We then repeated the transwell assay using YAP shRNA#2 (another shRNA targeting a different YAP sequence from YAP shRNA#1). YAP shRNA#2‐transfected H2030‐BrM3 cells had decreased invasion ability compared to control shRNA‐transfected H2030‐BrM3 cells (*P* < .01) (Figure 4E,F). The cell viability of H2030‐BrM3 was assayed after YAP shRNA transfection (Figure S3D,E). Our results verified that YAP shRNA transfection indeed decrease migration and invasion abilities in H2030‐BrM3 and not by the effects of cell death.

We next investigated whether increased YAP expression promotes metastatic potential in parental H2030 cells. In H2030 cells transfected with YAP plasmid, YAP protein and mRNA expression were increased compared to H2030 cells transfected with pcDNA 3.1 (Figure S2A,B). YAP plasmid‐transfected H2030 cells also had increased migration and invasion abilities compared to pcDNA 3.1‐transfected H2030 cells in migration and transwell assays (Figure S2C‐F).

### 3.5 Inhibition of YAP by shRNA suppresses brain metastatic ability of H2030‐BrM3 cells in vivo

**Figure 5:**
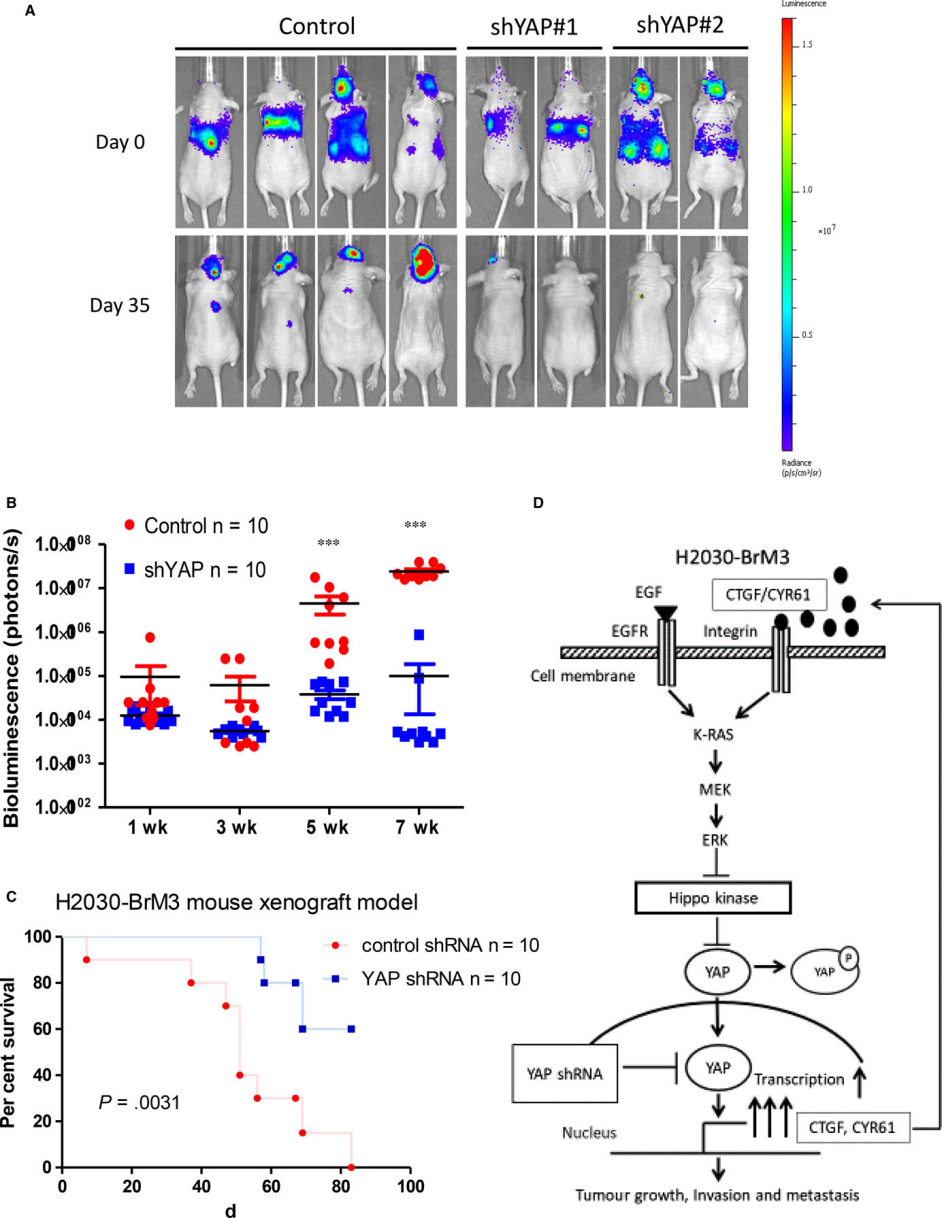
Inhibition of YAP by shRNA suppressed metastatic ability of H2030‐BrM3 cells in a murine model (control shRNA n = 10, YAP shRNA#1 n = 5, YAP shRNA#2 n = 5). A, Bioluminescence images of mice inoculated with control shRNA‐transfected H2030‐BrM3 cells, YAP shRNA#1‐transfected and YAP shRNA#2‐transfected H2030‐BrM3 cells. B, Tumour metastasis burden in control mice and mice inoculated with YAP shRNA‐transfected H2030‐BrM3 cells based on photon flux (photons per second) (***P ≤ .001). C, Kaplan‐Meier survival curves for control mice and mice inoculated with YAP shRNA‐transfected H2030‐BrM3 cells. D, Summary of the hypothetical model of our study. In H2030‐BrM3 cells, activation of YAP occurs at transcription mRNA level and increases YAP protein expression and mRNA expression of downstream genes CTGF and CYR61. CTGF/CYR61 forms an autocrine loop to activate the MAPK signalling pathway in H2030‐BrM3 cells. The enhancement of the MAPK signalling pathway increases the invasion and migration abilities and metastatic potential of H2030‐BrM3 cells

To investigate whether YAP inhibition suppresses the metastatic ability of H2030‐BrM3 cells, control shRNA‐transfected and YAP shRNA#1‐transfected H2030‐BrM3 cells were injected into nude mice. We then repeated the in vivo experiment using YAP shRNA#2. We found that the metastatic ability of H2030‐BrM3 cells in this murine model decreased significantly after YAP knockdown by shRNA, as shown in bioluminescence images (*P* < .001) (Figure 5A,B). Mice inoculated with YAP shRNA‐transfected H2030‐BrM3 cells survived significantly longer than mice inoculated with control shRNA‐transfected H2030‐BrM3 cells (*P* < .01) (Figure 5C).

### 3.6 Inhibition of YAP by shRNA decreased nucleus YAP immunohistochemistry stain of mouse brain tumour tissues

**Figure 6:**
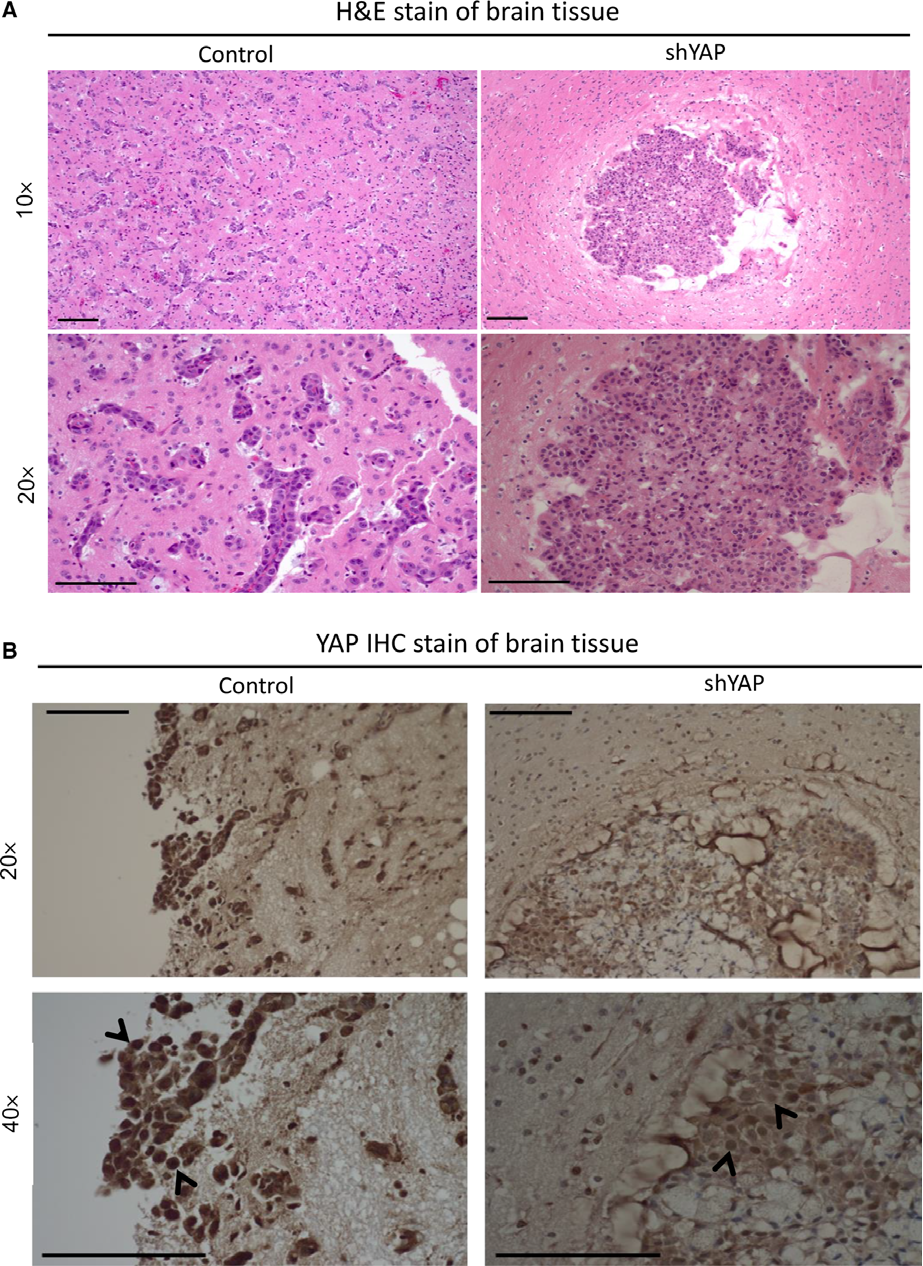
Histological analysis of mice brain tissue. A, H&E stain of brain tissues from control mice and mice inoculated with YAP shRNA‐transfected H2030‐BrM3 cells. B, YAP IHC stain of brain tissues from control mice and mice inoculated with YAP shRNA‐transfected H2030‐BrM3 cells. The scale bar = 100 μm

Brain tissues were collected from control and YAP shRNA groups for histological analysis after mice were killed. Haematoxylin and eosin (H&E) stain showed tumour metastasis within brain tissues (Figure 6A). YAP IHC staining was performed to see the late effect of shRNA transfection. The results showed that YAP IHC staining decreased in YAP shRNA brain tissues compared to control brain tissues (Figure 6A). Our result shows that inhibition of YAP by shRNA decreased nucleus YAP IHC stain of mouse brain metastatic tumour tissues.

## 4 DISCUSSION

Our study provides several lines of evidence that suggest the Hippo/YAP signalling pathway is important in promoting metastasis of lung adenocarcinoma. First, we found that H2030‐BrM3 (K‐ras^G12C^ mutation) and PC9‐BrM3 (EGFR^Δexon19^ mutation) cells have decreased p‐YAP(S127)/YAP protein expression ratio compared to parental H2030 and PC9 cells. Second, H2030‐BrM3 cells have increased YAP mRNA, expression of downstream genes CTGF and CYR61, and YAP immunofluorescence staining compared to parental H2030 cells. Finally, direct inhibition of YAP by YAP shRNA decreased the metastatic ability of H2030‐BrM3 cells in a murine model.

According to recent studies, YAP takes over K‐ras as a cancer driver in NSCLC.^23^, ^24^, ^25^ A previous study also identified that some NSCLC cell lines harbouring K‐ras mutations were not K‐ras‐dependent cells, and that for those cell lines, K‐ras may be not a therapeutic target.^22^ Moreover, H2030 cells (K‐ras^G12C^ mutation) are not responsive to therapies targeting K‐ras, mitogen‐activated protein (MAP)‐ERK kinase (MEK) and the extracellular signal‐regulated kinase (ERK) signalling pathway.^29^, ^30^, ^31^, ^32^ In our study, inhibition of K‐ras by K‐ras siRNA in H2030‐BrM3 cells did not decrease YAP protein expression, which suggests these cells may not be K‐ras‐dependent. Therefore, we investigated YAP as a therapeutic target for metastasis of H2030‐BrM3 cells because they have increased YAP mRNA and YAP protein expression.

Another metastatic lung adenocarcinoma cell line PC9‐BrM3 (EGFR^Δexon19^ mutation) was derived from PC9 cells (EGFR^Δexon19^ mutation) with a known EGFR exon 19 deletion mutation.^28^, ^31^, ^32^ Currently, erlotinib is effective in treating metastatic lung adenocarcinoma harbouring EGFR exon 19 deletion mutation.^9^, ^33^, ^34^, ^35^, ^36^ Our results showed that YAP protein expression decreased after erlotinib treatment and inhibition of EGFR by EGFR siRNA in PC9‐BrM3 cells. We recently reported crosstalk between Hippo/YAP and EGFR/extracellular signal‐regulated kinase signalling pathways in human NSCLC cells.^19^, ^21^ Collectively, PC9‐BrM3 cells are dependent on the EGFR signalling pathway and inhibiting that pathway can inhibit the YAP signalling pathway and control metastasis of PC9‐BrM3 cells.

Yes‐associated protein transcription can be stimulated by SIRT1,^37^ and SIRT1 is highly expressed in NSCLC brain metastasis tissues.^38^ Together, these finding may partially explain the increase in YAP mRNA expression level in H2030‐BrM3 cells as shown in our study. Much work is needed to verify the source of increased YAP transcription in metastatic NSCLC.

The activation of YAP forms autocrine loops with the ERBB pathway to promote cancer progression, migration and invasion in various cancers.^39^, ^40^, ^41^, ^42^ Moreover, Hippo downstream genes CTGF and CYR61 have been reported to activate integrin‐mitogen‐activated protein kinase (MAPK) and the AKT signalling pathway and promote cancer progression and metastasis in cancer.^43^, ^44^, ^45^, ^46^ MAPK and the AKT pathways negatively regulate Hippo kinase and reduce p‐YAP (S127).^47^, ^48^ The decreased p‐YAP(S127)/YAP ratio indicates an increase in YAP stability, and then more YAPs enter nucleus to activate downstream gene expression. These findings may explain our finding that YAP mRNA level increased modestly (about 1.5‐fold), but the reduction in p‐YAP was more prominent. The mechanism of high metastatic potential in H2030‐BrM3 cells is summarized in Figure 5E.

In our study, YAP protein expression decreased more than 70% after YAP knockdown by shRNA and siRNA, but the decrease in GTIIC reporter activity and CTGF mRNA expression was not as great as the decrease in YAP protein in our in vitro experiments. Actually, YAP does not account for all the GTIIC reporter activity and other mediators exist.^49^ Like GTIIC reporter activity, CTGF mRNA expression did not decrease as markedly as YAP protein after shRNA and siRNA transfection in H2030‐BrM3 cells because YAP is one of several factors that control CTGF expression; for example, TGF‐β can control CTGF expression.^50^, ^51^

Serpin I1 (neuroserpin) is an important regulator in promoting brain metastasis of lung adenocarcinoma H2030‐BrM3 cells in murine models. Serpin I1 helps cancer cells to infiltrate brain by inhibiting plasminogen activator (PA)‐plasmin system.^27^ In our study, sequence analysis of serpin I1 revealed a putative YAP binding site more than 6 kb upstream of the transcription start site, and it is known that YAP can bind distal enhancers through TEAD to control transcription of downstream genes.^52^ Using a ChIP assay, we showed that YAP binds to the serpin l1 enhancer in H2030‐BrM3 cells, and inhibiting YAP by YAP shRNA and YAP siRNA decreased serpin I1 protein and mRNA expression in H2030‐BrM3 cells. These results suggest that YAP signalling may be involved in regulating serpin I1, and through inhibition of YAP can partly suppress serpin I1 expression and then decrease the potential of brain metastasis in H2030‐BrM3 cells. These results also explained that inhibition of YAP by shRNA did not suppress proliferation ability of H2030‐BrM3 cells markedly, but brain metastasis occurred late in the in vivo experiment of shRNA group.

To our knowledge, our study is first to provide the key evidence that direct inhibition of YAP by shRNA suppresses lung adenocarcinoma H2030‐BrM3 cell metastasis in a murine model. This is one the best metastasis models that are currently available using human lung cancer cells. Most unresectable lung cancer patients (e.g., stage IV lung cancer) are at stage of colonization, and the treatment options for these patients are very limited (e.g., TKI and immunotherapy). Targeting this stage can be beneficial to patients with late‐stage lung cancer, although we also wish to find a better metastasis models (e.g., orthotopic models) that can help us to validate whether YAP is indeed a driver for the entire metastasis process (as suggested in in vitro transwell experiments).^6^, ^26^, ^27^, ^53^

Our study shows that YAP plays a key role in promoting brain metastasis in lung adenocarcinoma H2030‐BrM3 cells and that inhibition of YAP can suppress brain metastasis in vivo. These findings may have implications for future potential therapeutic strategies for K‐ras mutant metastatic lung adenocarcinoma. More studies to validate YAP as a therapeutic target for K‐ras mutant human metastatic lung adenocarcinoma are warranted.

## CONFLICT OF INTERESTS

All authors have no conflict of interests.

## AUTHOR CONTRIBUTIONS

Conception and design: PC. Hsu and L. You; development of methodology: PC. Hsu, J. Miao, Z. Huang, Z. Xu, J. You, YL. Yang and G. Chan; acquisition of data (provided animals, provided facilities, etc.): PC. Hsu, J. Miao, Z. Huang, CC. Yeh, S. Liu and D.M. Jablons; analysis and interpretation of data (pathologic analysis): A. Urisman; writing, review and/or revision of the manuscript: PC. Hsu, CT. Yang and L. You; administrative, technical or material support (organizing data): PC. Hsu, Z. Xu and L. You; and study supervision: L. You.
