# Supplementary figures and images for: Inhibition of yes‐associated protein suppresses brain metastasis of human lung adenocarcinoma in a murine model
Source: J Cell Mol Med. 2018 Mar 24;22(6):3073–85. doi: 10.1111/jcmm.13582 (PMC5980132; doi:10.1111/jcmm.13582)

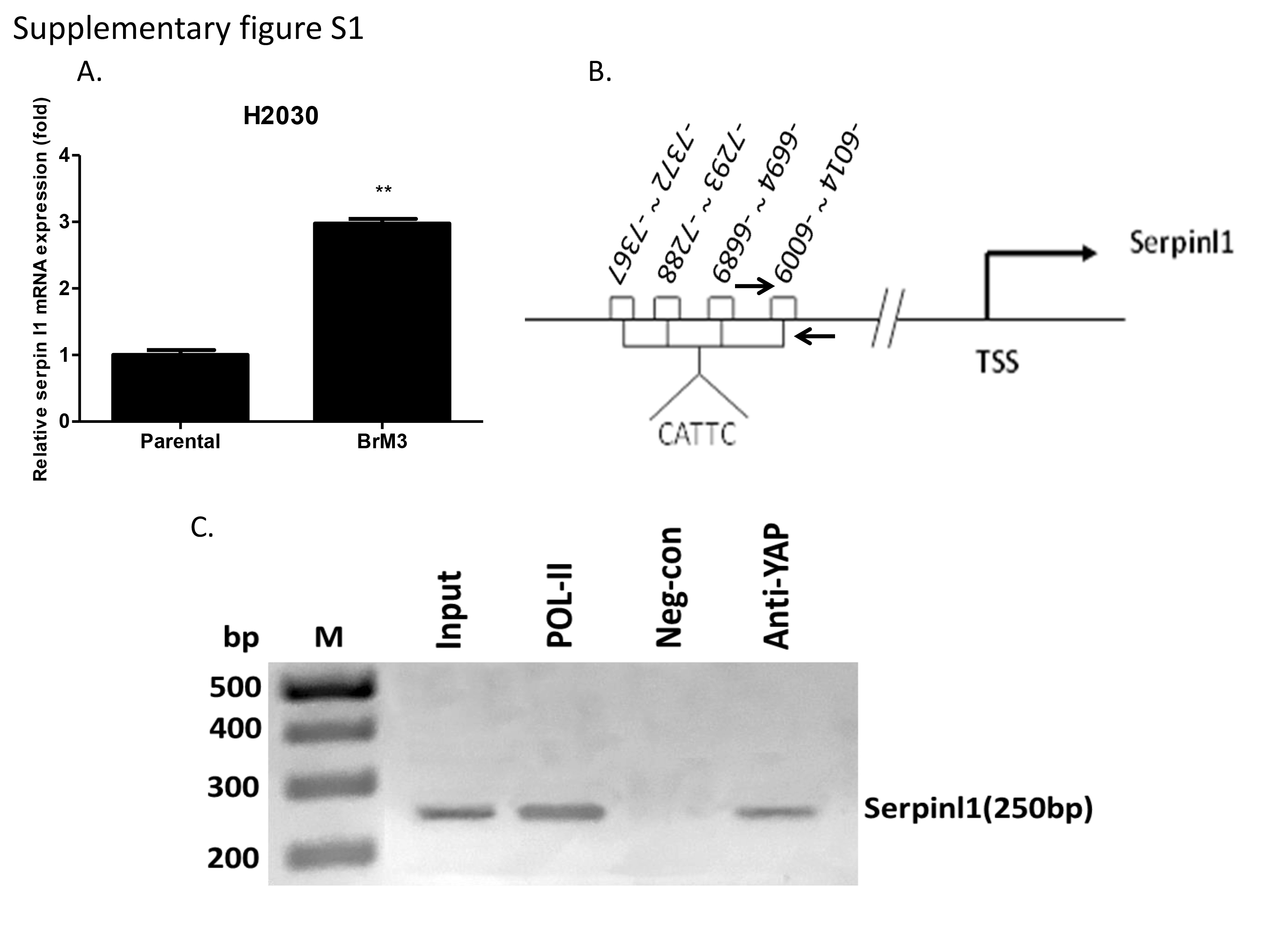

Supplement: Supplementary file 1 [file JCMM-22-3073-s001.png]

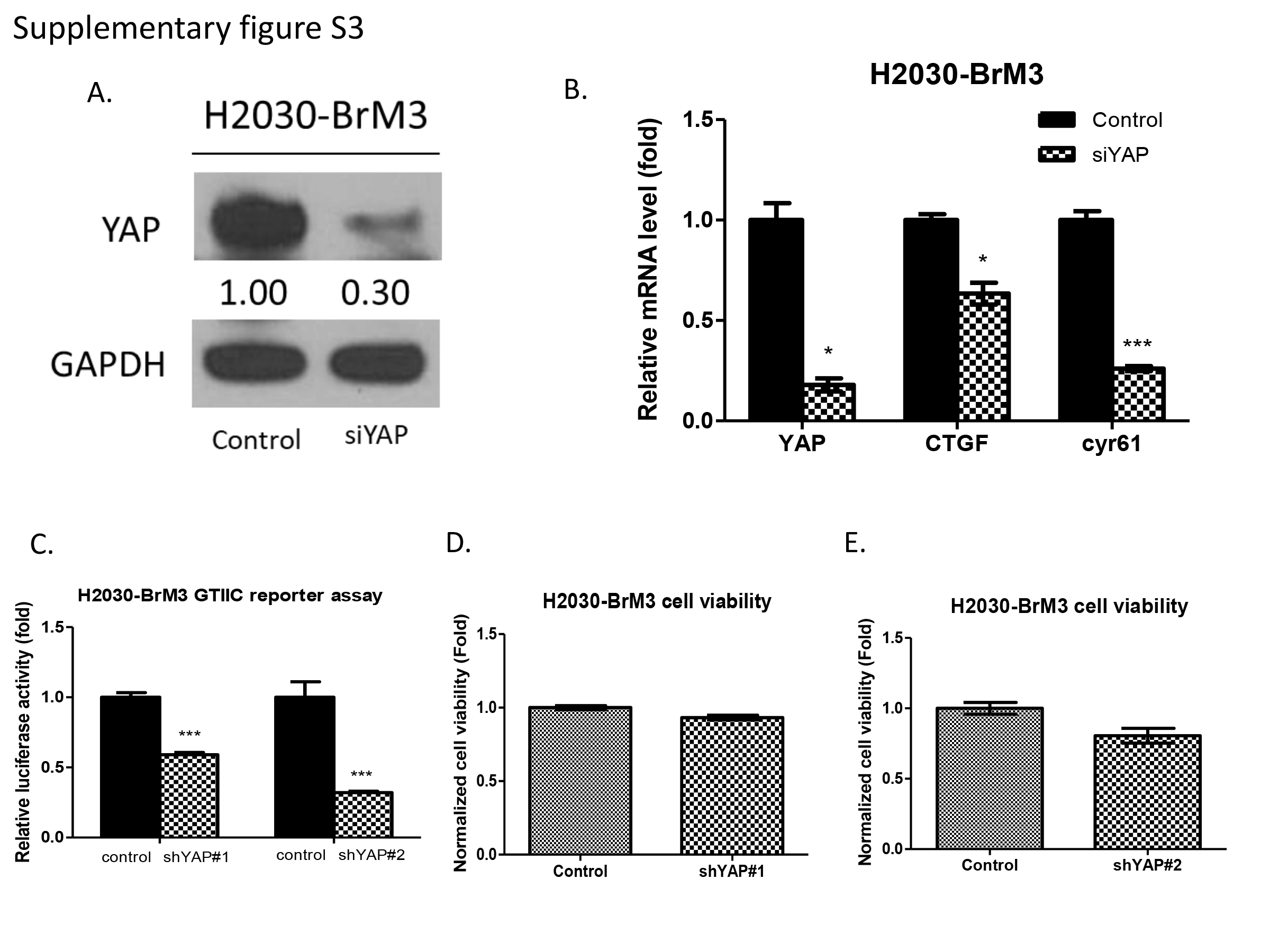

Supplement: Supplementary file 2 [file JCMM-22-3073-s002.png]

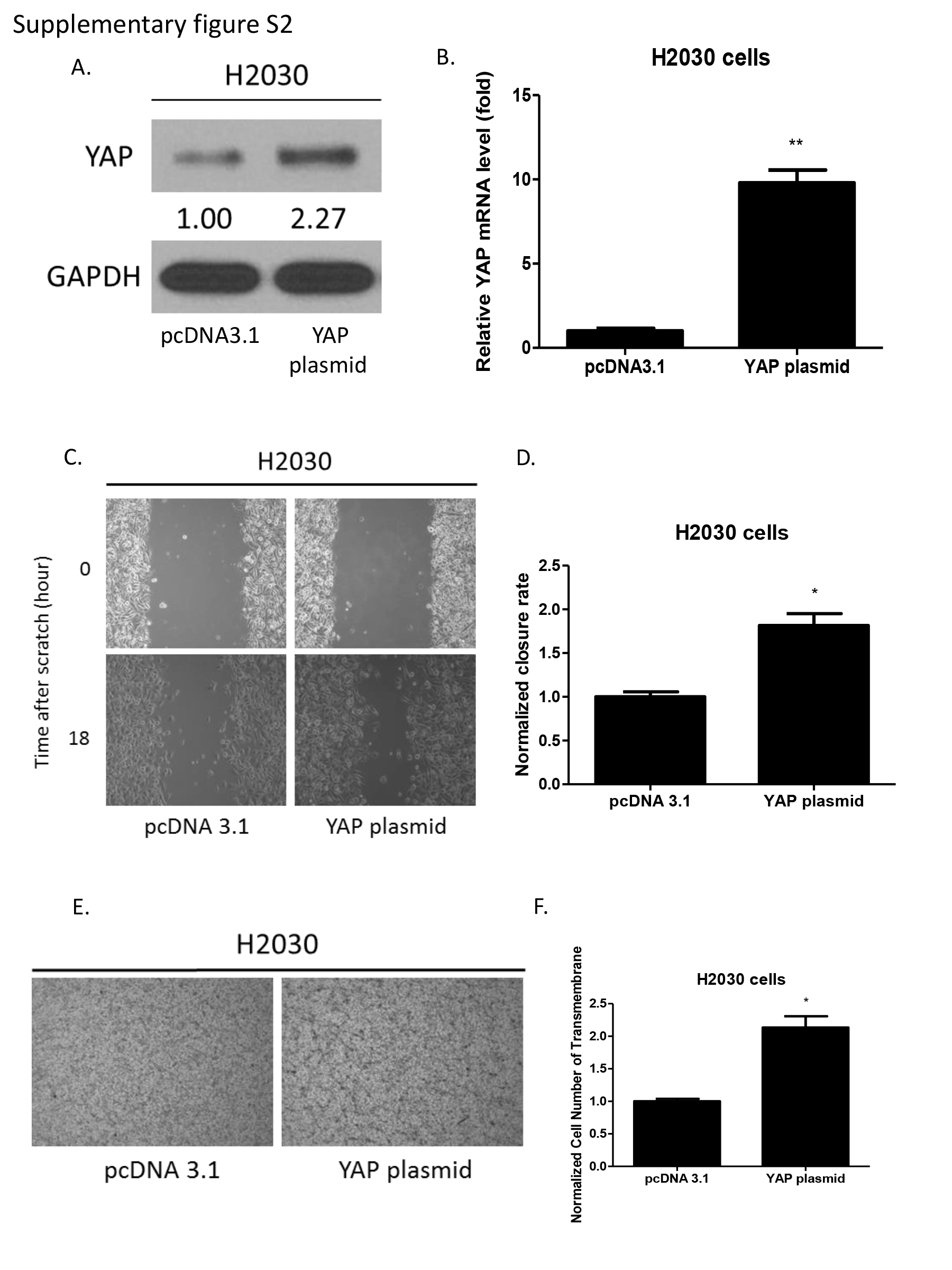

Supplement: Supplementary file 3 [file JCMM-22-3073-s003.png]
